# Shoulder pain, shoulder disability, and depression as serial mediators between stress and health-related quality of life among middle-aged women

**DOI:** 10.1186/s12955-022-02054-1

**Published:** 2022-10-13

**Authors:** Jihyun Oh, Myung Kyung Lee

**Affiliations:** 1 Department of Nursing, College of Nursing and Health, Kongju National University, 32588 Kongju, South Korea; 2 College of Nursing, Research Institute of Nursing Science, Kyungpook National University, 41944 Daegu, South Korea

**Keywords:** Shoulder pain, Shoulder disability, Depression, Stress, Health-related quality of life

## Abstract

This study analyzed the mediating effects of shoulder pain, disability, and depression on the relationship between stress and health-related quality of life among middle-aged women using a serial mediation model. **Methods.** Data on stress, health-related quality of life, shoulder pain, shoulder disability, and depression were collected from 565 women aged 35–64 years living in Seoul, South Korea, from May 13 to 23, 2021, using a self-reported, structured survey. SPSS PROCESS macro (Model 6) and serial mediation analysis were used to analyze the relationship between stress and health-related quality of life among participants, with shoulder pain, shoulder disability, and depression as mediators. **Results.** The results indicate that stress had a statistically direct impact on health-related quality of life. In the serial mediation analysis, shoulder pain, disability, and depression were found to be statistically significant, thus affecting the relationship between stress and health-related quality of life, with an explanatory power of 33%. Therefore, the relationship between stress and health-related quality of life was partially mediated by these variables. **Conclusions.** Thus, this study suggests the need for healthcare workers to develop methods, such as exercise intervention programs based on various degrees and types of physical activity, to improve health-related quality of life and reduce stress caused by shoulder pain, shoulder disability, and depression among middle-aged women.

## Background

Shoulder pain is the third most common musculoskeletal complaint among adults, with a lifetime incidence of ≥ 60% [1, 2]. It was found to persist for ≥ 12 months after the first onset in ≥ 60% of participants [3, 4]. Furthermore, it often becomes a chronic condition that makes life difficult and uncomfortable. It also causes functional impairment owing to degenerative changes in tissues and reduces patients’ health-related quality of life (HRQoL) [5].

Recently, the prevalence of shoulder pain has been observed in people in their 30s, and more commonly in those over 40 years of age. Several diseases such as adhesive capsulitis, rotator cuff tendinopathy, glenohumeral osteoarthritis, and neurological and vascular diseases cause shoulder joint problems. In general, women have lower muscle strength and athletic ability than men, especially because relaxation of their muscles is affected by female hormones, thus making them more prone to musculoskeletal diseases, including shoulder pain [6–8].

Shoulder pain and disability lower quality of life [9, 10]. An individual’s assessment of satisfaction with the physical, mental, social, and psychological aspects of life is defined as quality of life [11]. Assessing HRQoL to prevent diseases and promote health has gained increased attention in health-related research and practice [12].

Previous studies have reported a significant correlation between women and shoulder pain; however, such disabilities have not been fully addressed [13–16]. Previous studies have shown that functional disability is a significant factor influencing depression in older women [17]; however, few studies have focused on the relationship between shoulder pain, disability, and depression among middle-aged Korean women. Therefore, it is necessary to understand the relationship between shoulder pain, disability, and HRQoL in middle-aged Korean women.

A previous study reported that living with chronic pain correlates with and causes psychological stress [18]; therefore, people who have had negative experiences in life have a high risk of developing chronic musculoskeletal pain [19, 20]. In particular, the degree of perceived pain is high in older people and in those with higher stress levels [21]. Therefore, it is necessary to identify whether stress in middle-aged Korean women directly affects shoulder pain and to analyze how this relationship affects HRQoL.

Previous studies have shown that perceived stress is a known risk factor for chronic shoulder pain [22, 23] and that stress negatively affects quality of life [24]. Limited physical function [25] and depression [26] have been reported to lower the quality of life. Furthermore, depression is more common in patients with musculoskeletal disease, especially among those who find it difficult to perform daily activities [25]. Therefore, this study investigated the relationships between stress and shoulder pain, shoulder pain and disability, shoulder disability and depression, and depression and HRQoL.

According to previous studies, stress was correlated with pain [27, 28], disability [29], depression [30–32], and quality of life [33]. Pain was correlated with disability [34, 35], depression [36], and quality of life [34, 37]. Disability was correlated with depression and quality of life [34, 37–40]. Depression is correlated with quality of life [41]. According to the with or without concomitant depression, there were differences in symptoms, functioning, and quality of life in women with musculoskeletal pain.

**Fig. 1:**
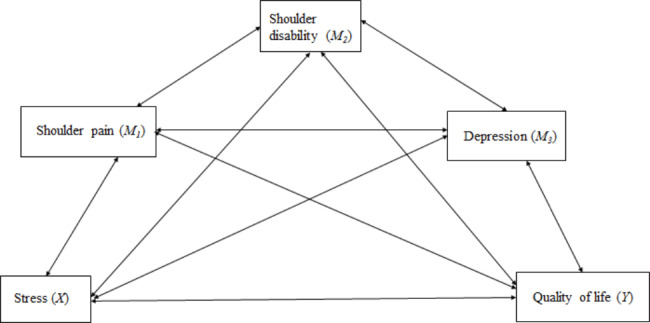
A conceptual framework of the study

**Fig. 2:**
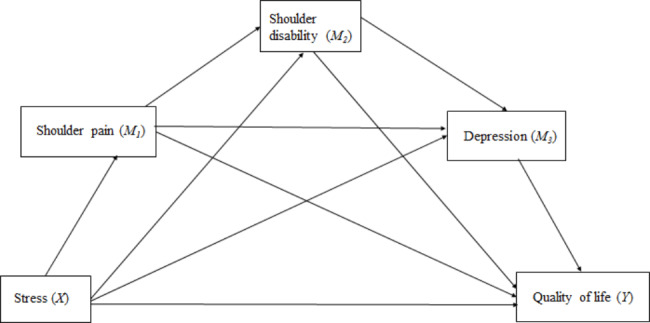
A hypothetical model of the study

These correlations, based on previous literature, are presented in Fig. 1 as a conceptual framework for the study. However, this study tested the causal relationship among stress (X), shoulder pain (M1), shoulder disability (M2), depression (M3), and quality of life (Y), as shown in Fig. 2, as a hypothetical model.

The transactional model of stress and coping, proposed by Lazarus and Folkman, describes stress pathways [42–44]. Their transactional model provides a theoretical framework for this study. The model suggests that the stress response is highly influenced by individual appraisal processes. Appraisals are believed to have an impact on the coping strategies chosen by individuals. Coping affects the immediate stress response as well as long-term health, psychological well-being, and social functioning (X↔M1, X↔M2, X↔M3, X↔Y) [44]. The pathways between stressors and stress response (stress) and long-term health, psychological well-being, and social functioning can be applied to the relationship between stress and health status (i.e., pain, disability, depression, and quality of life) proposed in this study. According to Lipowski [45] and the self-regulatory model of Leventhal et al. [46], cognitive representations of subjects with their own illnesses determine the coping behaviors adopted and, consequently, the illness outcome.

Based on previous research, this study identified the relationship between stress and HRQoL in middle-aged women and proposed a serial multiple mediation model to understand this relationship. It further investigated how stress directly and indirectly affects HRQoL through mediators, such as shoulder pain, shoulder disability, and depression. When these predicted relationships were determined, we validated the serial multiple mediation model to determine the extent to which each variable affected others. Overall, this study aimed to examine the effect of stress on HRQoL among middle-aged women. Our hypotheses were as follows: Hypothesis 1: Stress is negatively associated with quality of life among middle-aged women. Hypothesis 2: Shoulder pain plays a mediating role in the association between stress and quality of life. Hypothesis 3: Shoulder disability plays a mediating role in the association between stress and quality of life. Hypothesis 4: Depression mediates the association between stress and quality of life. Hypothesis 5: Shoulder pain, disability, and depression play a serial mediation role in the association between stress and quality of life.

## Methods

This cross-sectional survey examined the relationship between stress and HRQoL in middle-aged women. All participants were recruited through personal contact with two community centers located in Seoul. Each community center had a monthly average of 500–1000 local residents. The researchers provided a questionnaire after obtaining informed consent with a voluntary decision before the participants entered the research. Data were anonymously collected from 589 women aged 35–64 years living in Seoul, South Korea. After considering the dropout rate, 565 participants were included in this study, ensuring an appropriate sample size. However, the common method bias in cross-sectional studies makes it difficult to accurately obtain an individual’s different sources and perceptions because of the use of consistent Likert-type scales.

Data were collected on May 13–23, 2021. The selection criteria for the study were as follows: (1) women aged 35–64 years and (2) ability to communicate and complete a self-report survey. The surveys were directly distributed to participants who provided written consent after being informed of the study purpose. In principle, the participants completed the questionnaire independently; they were supported by the research staff only if they required assistance.

### Measurement

#### Depression

The self-reported Patient Health Questionnaire (PHQ)-9, used in screening for depression, was developed by Kroenke et al. [47], and the Korean version, adapted by Park et al. [35], was adapted for this study. It comprises nine items corresponding to the diagnostic criteria for major depression, based on the *Diagnostic and Statistical Manual of Mental Disorders IV*. The PHQ-9 evaluates the frequency of these symptoms over a two-week period. The response of each tool was evaluated on a 4-point Likert scale as follows: 0 for “never,” 1 for “a few days,” 2 for “more than one week,” and 3 for “almost every day.” Kroenke et al. [47] used a score ranging from 0 to 27 points, with four break points of the PHQ-9 that divided the level of depression into five groups according to severity of symptoms (minimal, 0–4; mild, 5–9; moderate, 10–14; moderate-to-severe, 15–19; and severe, 20 or more). However, Park et al. [35] suggested that depressive disorders can be suspected when the total PHQ-9 score is > 5 points. Their study [35] had a Cronbach’s alpha of 0.81, whereas the current study had a Cronbach’s alpha of 0.85, indicating higher reliability.

#### Shoulder pain and disability index

The shoulder pain and disability index was developed by Roach et al. [48] to evaluate the degree of pain and disability and adapted by Seo et al. [49]. It comprises 13 questions and is divided into two supplementary scales that include five questions in the pain subscale to evaluate the degree of pain and eight questions in the function/disability subscale to evaluate the degree of upper limb disability when performing different activities of daily living. Thirteen items were evaluated on a 10-point Likert scale, with 0 indicating no pain and 10 indicating very severe pain. The results from each tool were converted to a 100-point scale. The higher the score obtained using this tool, the greater is the degree of shoulder pain, damage, and disability. The Cronbach’s alpha in this study was 0.94, indicating high reliability.

#### Stress

The extent to which an individual perceived a specific stressful condition was measured. The Korean Perceived Stress Scale (PSS) was used to measure perceived stress, as adapted by Lee et al. [50] from the PSS developed by Cohen et al. [51]. The tool comprises 10 items that are assessed using a 5-point Likert scale ranging from never (0 points) to very often (4 points). In Lee et al. [50], the Cronbach’s alpha of this tool was 0.88, whereas it was 0.96, indicating a higher reliability. The total points ranged from 0 to 40, where 0–13 indicated low level stress, 14–26 indicated normal level stress, and 27–40 indicated high level stress.

#### Health-related quality of life

The 12-Item Short Form Health Survey (SF-12) developed for the Medical Outcomes Study by Ware et al. [52] was used to evaluate health-related quality of life in this study. The tool was divided into eight subregions for two main parts: a physical component summary (PCS-12) and a mental component summary (MCS-12). It comprises 12 items, and the score of each sub-region was converted into a range of 0–100 points, regardless of the number of items, according to the specified calculation method of the SF-12. A higher score indicates a higher quality of life. The SF-12 is often used to assess the quality of life in relation to health, and its reliability and validity have been proven for all ages [53]. Cronbach’s alpha was 0.86, indicating high reliability.

### Ethical considerations

This study was conducted in accordance with the principles of the WMA Declaration of Helsinki and the study plan and process were approved by the Clinical Ethics Committee of Daejeon University. Written consent was obtained from all participants following a detailed explanation of the purpose and methods of the study before beginning the survey. Study participation was voluntary and participants could withdraw at any given time; the collected data were used for research purposes only. The guarantee of anonymity and autonomy of the participants was described. Survey data were stored in a secure cabinet.

### Statistical analyses

All study data were processed using SPSS for descriptive statistics and correlation analysis. The values indicated that the histogram and–a Q-Q plot normally distributed data were suitable for multivariate normal distribution from the population. We checked the normality assumption of the main variables using the Kolmogorov-Smirnov test and skewness and kurtosis tests [54]. Pearson’s correlation coefficient was used to examine the association between variables. Correlation analysis and mediatory variable analysis were conducted to determine the significance of the analysis because univariate and multivariate normality conditions were provided. Regression analysis was performed to verify the mediating effect using the PROCESS macro for SPSS [55].

Hayes’s PROCESS macro (Model 6) was used to understand the mechanism of shoulder pain, shoulder disability, and depression in the relationship between stress and HRQoL. In serial mediation, mediating factors (shoulder pain, disability, and depression) are expected to directly and indirectly affect HRQoL. The serial mediation model helps identify the precedence between the three mediating variables in the relationship between stress and HRQoL. In this model, the first mediating variable (shoulder pain) sequentially affected the second mediating variable (shoulder disability), which in turn affected the third mediating variable (depression). Bootstrapping of the SPSS PROCESS macro was used to test the mediating effects in this study [55]. Using random sampling, 10,000 samples were generated and a 95% bias-corrected confidence interval (BC CI) was employed to analyze the mediating effects [56]. If the 95% CI did not cover zero, the effect was considered significant.

## Results

### Participants’ general characteristics

The general characteristics of the participants are presented in Table 1. Of the 589 participants who answered the survey, 565 were included in the analysis, excluding those with incomplete responses and those who dropped out of the study (response rate: 95.9%). Depression levels measured using the PHQ-9 showed that minimal depression was the most common (39.6%), followed by mild depression (35.4%).

**Table 1:** Descriptive statistics of study population (*N* = 565)

Variable	N (%)	Mean (SD)
Mean Age (years) (range)		42.15 (6.37) (35–63)
Marital status		
Single	141 (25.0)	
Married	411 (72.7)	
Divorced & widowed	13 (2.3)	
Education		
≤High school	112 (19.8)	
≥College	453 (80.2)	
Exercise		
Yes	270 (47.8)	
No	295 (52.2)	
Smoking		
Yes	18 (3.2)	
No	547 (96.8)	
Alcohol		
Yes	265 (46.9)	
No	300 (53.1)	
Economic status		
≥Middle	470 (83.2)	
Low	95 (16.8)	
Sitting hours during work		4.06 (2.90)
Working hours per day		7.08 (2.77)
Chronic condition		
Without	363 (64.2)	
With	202 (35.8)	
Depression		
Minimal	224 (39.6)	
Mild	200 (35.4)	
Moderate	92 (16.3)	
Moderate-to-severe	39 (6.9)	
Severe	10 (1.8)	

### Scores for shoulder pain, shoulder disability, depression, stress, and health-related quality of life

Table 2 shows the overall average score for HRQoL, PCS-12, MCS-12, depression, stress, shoulder pain, and shoulder disability. The average score for depression was 6.73 (5.14), which was ≥ 5 points according to the overall average, suggesting depressive disability.

**Table 2:** Scores for shoulder pain, shoulder disability, depression, stress, and health-related quality of life (*N* = 565)

Variables	Min–Max	Mean (SD)
Health-related quality of life	25–91.07	66.28 (10.00)
PCS-12	23.08–88.46	65.28 (10.09)
MCS-12	20–100	67.15 (11.88)
Depression	0–27	6.73 (5.14)
Stress	4–38	18.99 (5.47)
Shoulder pain	0–100	42.68 (25.06)
Shoulder disability	0–100	26.82 (23.54)

Note. SD, standard deviation; PCS, physical component score; MCS, mental component score

### Correlations between shoulder pain, shoulder disability, depression, stress and health-related quality of life

The results were obtained by analyzing the correlations between the variables. HRQoL was negatively correlated with stress (r = − 0.249, p < 0.001), depression (r = − 0.261, p < 0.001), shoulder pain (r = − 0.103, p = 0.015), and shoulder disability (r = − 0.234, p < 0.001). Thus, the higher the stress, the worse the depression, and the more severe the shoulder pain and disability, the lower the HRQoL. Stress positively correlated with depression (r = 0.589, p < 0.001), shoulder pain (r = 0.221, p < 0.001), and shoulder disability (r = 0.228, p = 0.004). Depression positively correlated with shoulder pain (r = 0.357, p < 0.001) and shoulder disability (r = 0.366, p < 0.001). A positive correlation was found between shoulder pain and disability (r = 0.694, p < 0.001). Thus, the higher the stress, the worse the shoulder pain and disability and the higher the depression level. Moreover, when shoulder pain and disability were severe, the depression level was also high, and the worse the shoulder pain, the higher the shoulder disability.

### Mediating effects

**Fig. 3:**
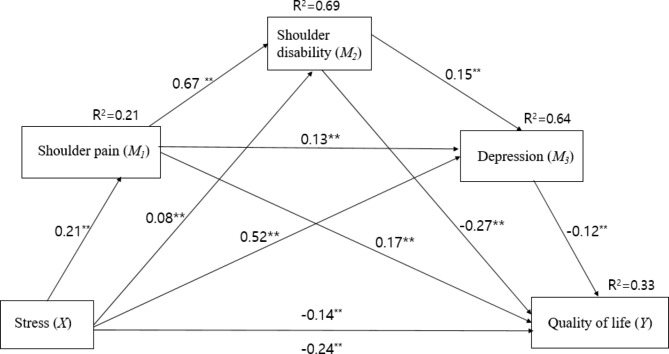
Results of multiple mediation model. ** p < 0.01

Table 3; Fig. 3 illustrate the findings of the tested model and how shoulder pain, disability, and depression mediate the relationship between stress and HRQoL in middle-aged women. Notably, there was a significant total effect (B = − 0.4408, SE = 0.0773, t = − 5.7020, p < 0.001) and direct effect (B = − 0.2598, SE = 0.0934, t = − 2.7816, p = 0.0056) of stress were found on HRQoL. The impact of stress on HRQoL was reduced when shoulder pain, shoulder disability, and depression were removed, but became significant when they were added to the model as mediators. The effect of the serial multiple mediation model was found to be statistically significant in predicting HRQoL based on shoulder pain, disability, and depression, and the explanatory power of the variables for HRQoL was 33% (R^2^ = 0.33, F = 16.671, p < 0.001).

The bootstrapped indirect effect of stress on HRQoL via shoulder pain, shoulder disability, and depression was significant (B = − 0.1809, SE = 0.0655, 95% BC CI [− 0.3157–0.0558]). The indirect effects of stress through mediators and the direct effects of stress were both found to be significant, indicating a partial mediating effect of shoulder pain, shoulder disability, and depression.

**Table 3:** Total, direct, and indirect effects for multiple mediation model (*N* = 565)

	Effect	SE	*t*	*P*	95% BC CI
Total effect of stress on health-related quality of life	−0.4408	0.0773	−5.7020	< 0.001	[− 0.5926, − 0.2889]
Direct effect of stress on health-related quality of life	−0.2598	0.0934	−2.7816	0.0056	[− 0.4433, − 0.0763]
Total indirect effect	−0.1809	0.0655			[− 0.3157, − 0.0558]
Indirect effect via shoulder pain	0.0679	0.0276			[0.0217, 0.1289]
Indirect effect via shoulder disability	−0.0414	0.0211			[− 0.0896, − 0.0059]
Indirect effect via depression	−0.1197	0.0574			[− 0.2368, − 0.0127]
Indirect effect via shoulder pain and shoulder disability	−0.0733	0.0215			[− 0.1199, − 0.0363]
Indirect effect via shoulder pain and depression	−0.0065	0.0044			[− 0.0170, − 0.0002]
Indirect effect via shoulder disability and depression	−0.0029	0.0023			[− 0.0087, 0.0001]
Indirect effect via shoulder pain, shoulder disability, and depression	−0.0050	0.0033			[− 0.0132, − 0.0003]

Note. BC CI, bias-corrected confidence interval

## Discussion

This study evaluated the extent to which the underlying mechanisms of shoulder pain, disability, and depression mediate the relationship between stress and HRQoL in middle-aged women. Consistent with our conceptual framework, it was determined that: (1) stress negatively correlated with HRQoL; (2) shoulder pain, shoulder disability, and depression mediated the relationship between stress and HRQoL; and (3) these mediators had indirect effects on HRQoL in a sequential manner.

Similar to previous studies, our data also showed a close relationship between stress and HRQoL [57, 58]. Stress is a risk factor for poor physical and mental health [59]. Middle-aged Korean women experience more stressful events than men, as they perform different social roles within their families, including those related to pregnancy, giving birth, and childcare, and experience events such as separation, divorce, job loss, and the disease or death of family members. They often perform repetitive and unpaid household duties in addition to a fixed duration of working hours; therefore, compared to men, they are more vulnerable to stressors that threaten their social and daily lives [60]. Social support at work and home can positively impact an individual’s physical and mental health and reduce stress [61, 62]. Therefore, systematic family and social support should be provided to improve HRQoL and to reduce and control stress in middle-aged women.

In this study, stress had direct and indirect effects on HRQoL through shoulder pain and disability. Psychosocial factors, such as stress or anxiety, further activate pain perception [63, 64]. Shoulder pain often persists even after recovery [65] and, sometimes becomes a chronic condition (lasting for more than three months). It may occur in isolation or may be accompanied by neck or upper-back pain [66]. Humans can perceive stress and modify their responses accordingly. Therefore, to reduce shoulder pain or disability caused by stress, it is important to increase adjustment to stress and the threshold to endure it. As chronic pain and stress can be managed and controlled by proper exercise [67], it is necessary to conduct an intervention study in the future to control stress and pain by integrating exercise into the lives of middle-aged women.

This study showed that stress has direct and indirect effects on HRQoL, mediated by shoulder pain, disability, and depression. These results are consistent with those of previous studies [65, 68], indicating that the higher the depression, the lower the HRQoL, and the higher the pain, the higher the depression and lower the HRQoL [69]. The results of this study suggest that stress directly affects HRQoL and causes shoulder pain, shoulder disability, and depression, all of which have direct and indirect effects on HRQoL. Therefore, controlling and managing stress is important for improving the HRQoL. Stress has a secondary effect on physical symptoms, such as pain and disability, and mental symptoms, such as depression, as well as a negative effect on the quality of life.

Therefore, interventions that prevent and control stress caused by various complex factors experienced by middle-aged women should consider improving their physical and mental health. A previous study [67] reported that stress can be controlled through exercise and physical activity. Exercise has been reported to improve shoulder pain, shoulder disability, depression, and HRQoL [70, 71]. Therefore, future studies should develop an intervention program that includes various degrees and types of physical activity to reduce the stress experienced by women in their daily lives, restore their physical health if they have symptoms of physical health conditions and disabilities, and manage mental health conditions, such as depression, thereby helping and supporting them in a healthy and happy life.

### Limitations

This study had several limitations. First, only participants living in Seoul, South Korea were included using convenience sampling. Therefore, the results of this study cannot necessarily be extrapolated to middle-aged women in other countries. Second, this study analyzed the relationship between stress, HRQoL, shoulder pain, shoulder disability, and depression using a self-report survey. Therefore, the cause-and-effect interpretations of these variables cannot be established. Future studies should analyze and interpret data using tools to perform a more detailed and objective evaluation of the various factors that affect HRQoL in middle-aged women. Despite these limitations, this study provides new insights into the relationship between stress and HRQoL and analyzes the degree of direct and indirect effects of shoulder pain, shoulder disability, and depression on stress. Furthermore, this study suggests that improving the HRQoL of middle-aged women and reducing stress necessitate the development of interventions to reduce and manage shoulder pain and disability, and reduce the depression caused by these physical symptoms.

## Conclusion

This study analyzed the relationship between stress and HRQoL using serial mediation analysis. Therefore, the direct effect of stress on HRQoL was statistically significant. Additionally, according to the serial mediation analysis, the indirect effect of stress on HRQoL through shoulder pain, shoulder disability, and depression was found to be statistically significant, and the explanatory power of these variables affecting HRQoL was 33%. Thus, the study results highlight the importance of stress in maintaining HRQoL and suggest the need to develop specific health promotion programs (e.g., exercise programs) to reduce and control stress. These results further suggest that healthcare workers should develop and implement exercise intervention programs to reduce stress, shoulder pain, shoulder disability, and depression in middle-aged women. It is certain that consistent efforts, including exercise intervention programs and their evaluations, will enable middle-aged women to lead healthy lives and improve their HRQoL.

## Declarations

### Availability of supporting data

The datasets generated and/or analyzed during the current study are not publicly available but are available from the corresponding author upon reasonable request.
